# Analysis of quantum key distribution based on unified model of sequential state discrimination strategy

**DOI:** 10.1038/s41598-024-60020-x

**Published:** 2024-05-04

**Authors:** Min Namkung, Younghun Kwon

**Affiliations:** 1 Center for Quantum Information, Korea Institute of Science and Technology (KIST), Seoul, 02792 Republic of Korea; 2 Department of Applied Physics, Hanyang University (ERICA), Ansan, 15588 Republic of Korea

**Keywords:** Quantum information, Quantum mechanics

## Abstract

The quantum key distribution for multiparty is one of the essential subjects of study. Especially, without using entangled states, performing the quantum key distribution for multiparty is a critical area of research. For this purpose, sequential state discrimination, which provides multiparty quantum communication and quantum key distribution for multiple receivers, has recently been introduced. Moreover, the sequential state discrimination is applicable for the security analysis against an eavesdropper’s attack. In this work, we provide the security analysis of quantum key distribution by proposing a unified model of sequential state discrimination including an eavesdropper. In this model, the success probability of eavesdropping is used as a figure of merit for the security analysis. Moreover, we obtain a non-zero secret key rate between the sender and receiver, which implies that the sender and receiver can share a secret key despite the eavesdropper’s scheme that optimizing the success probability of eavesdropping. Further, we propose an experimental methodology for the proposed model, which is implementable with linear optics. We observe that the secret key between the sender and receiver can be non-zero, even with imperfections.

## Introduction

Quantum physics restricts perfect detection of a physical system’s state, which contradicts the argument of classical physics^1–4^. This fact takes a major role of quantum state discrimination in quantum information processing. According to the optimal strategy of the quantum state discrimination required in terms of the figure of merit, there exist well-known strategies such as minimum error discrimination^5–14^, unambiguous discrimination^15–23^, maximal confidence^24^, and a fixed rate of inconclusive results^25–33^, which can be applied to two-party quantum communication.

There can be many receivers in quantum communication, and the strategy of the quantum state discrimination between two parties needs to be extended to multiple parties. In 2013, Bergou et al.^34^ proposed sequential state discrimination in which many parties can participate as receivers. The sequential state discrimination is process in which the post-measurement state of a receiver is passed to the next receiver. The fact that the probability for every receiver to succeed in discriminating the given quantum states is nonzero implies that all these receivers can obtain the information of the quantum state of the sender, from the post-measurement state of the preceding receiver^35–40^. It was shown that sequential state discrimination can provide multiparty B92 protocol^41^, which was implemented using quantum optical experiment^42,43^. this gives us that the sequential state discrimination can be exploited to construct a general quantum key distribution scenario and to analyze the security thereof.

**Figure 1:**
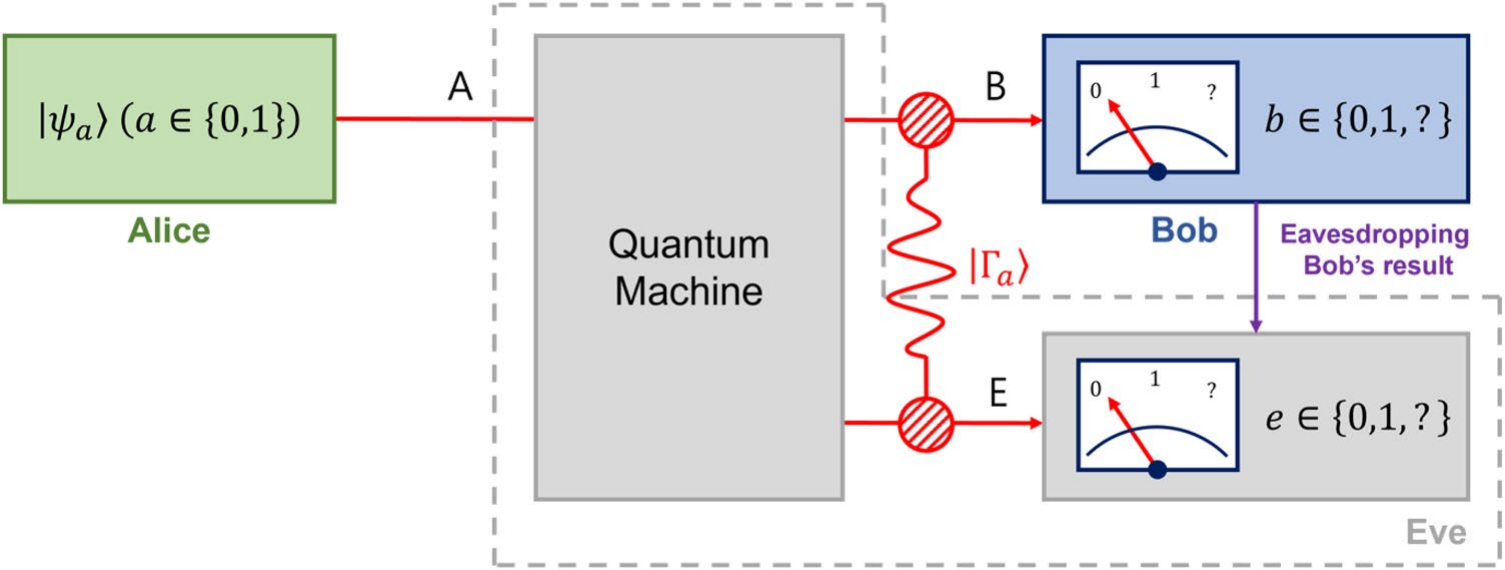
Type-I structure of Eve’s scheme for eavesdropping Bob’s measurement result. In this scheme, Eve uses a quantum machine that deterministically transforms Alice’s state $|\psi _a\rangle$ to a composite system $|\Gamma _a\rangle$ written in Eq. (3) such that $\textrm{Tr}_E\left( |\Gamma _a\rangle \langle \Gamma _a|\right)$ $=\Lambda ^{(A\rightarrow B)}(|\psi _a\rangle \langle \psi _a|)$. Then, she measures her subsystem to obtain information about Bob’s measurement result. If Eve is unnoticed by Alice and Bob, then the quantum channel between Alice and Bob is described as a depolarizing channel $\Lambda ^{(A\rightarrow B)}$ in Eq. (2)

When sequential state discrimination is performed, one can assume that an eavesdropper may exist. Suppose that Alice and Bob performs quantum communication and Eve tries to eavesdrop messages between them. The eavesdropper can have two ways for eavesdropping. The first situation is the case where Eve tries to eavesdrop on Alice’s quantum state, which was analyzed in^40^. The second situation is where Eve tries to eavesdrop on the result of Bob. Even though the second situation as well as the first one is a major threat to secure communication, the security analysis to this case has not been done yet. We further note that lots of prepare-and-measure QKD scenarios have been proposed by numerous researchers^44–48^, which includes not only high-dimensional DV-QKD protocols^49^ but also CV-QKD ones^50,51^, and the concern about the threat is reasonable in these scenarios. The prepare-and-measure QKD scenarios are considered to be practical since it does not require an entanglement between a sender and a receiver such as E92 and BBM92 protocols^52,53^.

In this paper, we focus on the second case, in which an intruder tries to eavesdrop on the result of a receiver. We provide a systematic security analysis from a unified model of sequential state discrimination including an eavesdropper. In this proposed model, the success probability of eavesdropping and the secret key rate^54^ can be considered as a figure of merit for the security analysis. Specifically, the figure of merit for Eve is the success probability of eavesdropping, but the figure of merit for Alice and Bob is the secret key rate. Our study shows that although Eve performs an optimal measurement for the success probability of eavesdropping, the secret key rate between Alice and Bob is not zero.

In addition, we propose an experimental scheme that implements a new sequential state discrimination method composed of Alice–Eve–Bob. This scheme consists of a linear optical system similar to a Sagnac interferometer^42,55^. The experimental setup can realize optimal success probability of eavesdropping for Eve, as well as non-zero secret key rate between Alice and Bob against the Eve. In other words, the protection of quantum communication between two receiters against an eavesdropper’s optimal scheme is possible with experimentally feasible setup. Further, we provide the success probability of eavesdropping and the secret key rate, considering the imperfections that can occur in the source, channel, and detector. White noise and colored noise are considered imperfections of the source^56^. The dark count rate and detection efficiency are considered imperfections of the detector^57^. We further propose that, despite these imperfections, the non-zero secret key rate between Alice and Bob is possible.

## Results

### Eavesdropper’s strategies

For an intruder, there are two ways of eavesdropping. The first is to eavesdrop on the quantum state of sender Alice and the other is to eavesdrop on the result of receiver Bob. When the intruder Eve, eavesdrops on the quantum state of sender Alice, she can do it using unambiguous discrimination, without an error. However, from the argument of sequential state discrimination, this process can be observed by Alice and Bob^40^. Therefore, the sender and receiver can recognize the presence of an eavesdropper.

When Eve wants to eavesdrop on the result of receiver Bob, she should be in a quantum entangled state with Bob. Assuming that the existence of an eavesdropper is unnoticed, the eavesdropping can be described as a noisy quantum channel to of Alice and Bob as Fig. 1a. When Alice prepares $|\psi _a\rangle$ ($a\in \{0,1\}$)

1 $$\begin{aligned} |\psi _a\rangle =\sqrt{\frac{1+s}{2}}|1\rangle +(-1)^a\sqrt{\frac{1-s}{2}}|2\rangle , \end{aligned}$$

with prior probability $q_a$, the noisy quantum channel between Alice and Bob can be described as follows:

2 $$\begin{aligned} \Lambda ^{(A\rightarrow B)}(|\psi _a\rangle \langle \psi _a|)_A=\eta _{AB}|\psi _a\rangle \langle \psi _a|_B+(1-\eta _{AB})\frac{\mathbb {I}_B}{2}. \end{aligned}$$

Here, the lower indices *A* and *B* denote the systems of Alice and Bob. $\mathbb {I}_B=|1\rangle \langle 1|+|2\rangle \langle 2|$ is an identity operator defined in the system of Bob, which consists of an orthonormal basis $\{|1\rangle ,|2\rangle \}$. In Eq. (2), $\eta _{AB}\in [0,1]$ denotes the channel efficiency between Alice and Bob.

**Figure 2:**
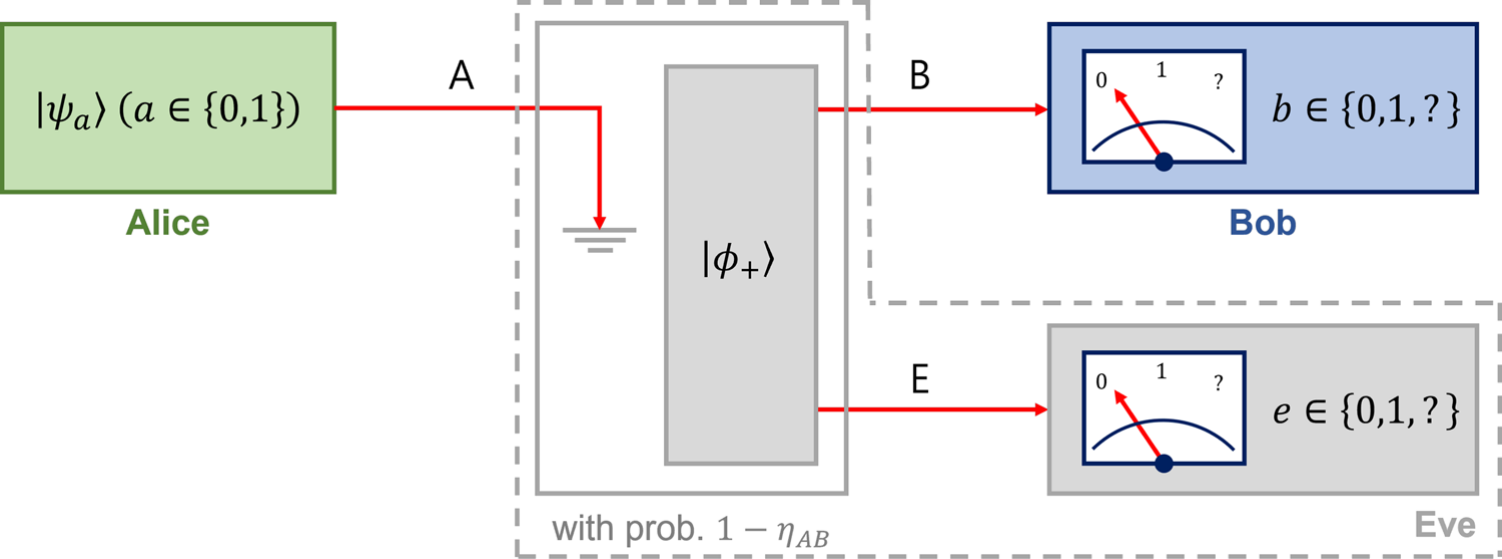
Type-II structure of Eve’s scheme. In this scheme, Eve discards Alice’s state $|\psi _a\rangle$ and shares a maximally entangled state $|\phi _+\rangle$ with Bob with a probability $1-\eta _{AB}$ as illustrated in the above figure, and lets the Alice’s state be transmitted to Bob with a probability $\eta _{AB}$.

#### Type-I structure of eavesdropper’s scheme

Let us consider the eavesdropper’s scheme illustrated as Fig. 1b. If quantum systems of Bob and Eve are considered, Eve uses a quantum machine to deterministically transform the Alice’s state $|\psi _a\rangle$ to a composite state between Bob and Eve:

3 $$\begin{aligned} |\Gamma _a\rangle _{BE}=\sqrt{\eta _{AB}}|\psi _a\rangle _B\otimes |0\rangle _E+\sqrt{1-\eta _{AB}}|\phi _+\rangle _{BE}, \end{aligned}$$

with an entangled state

4 $$\begin{aligned} |\phi _+\rangle _{BE}=\frac{1}{\sqrt{2}}(|11\rangle +|22\rangle )_{BE}, \end{aligned}$$

where is the entangled state between Bob and Eve. Then, Eve performs a quantum measurement on her system to discriminate Bob’s measurement result. If $\eta _{AB}$ is equal to one, then the composite state in Eq. (3) is a product state. Thus, Eve cannot obtain information by measuring her subsystem. Otherwise, Eve can obtain the information about Bob’s measurement result. We note that the partial state of Bob is equal to Eq. (2).

#### Type-II structure of eavesdropper’s scheme

The drawback of the eavesdropping scheme introduced above is that it requires a quantum machine deterministically producing $|\Gamma _a\rangle$. Since designing the quantum machine can be difficult, we further propose an alternative eavesdropping scheme. In this scheme, we can consider a composite state between Bob and Eve as follows:

5 $$\begin{aligned} \sigma _{a,BE}=\eta _{AB}|\psi _a\rangle \langle \psi _a|_B\otimes |0\rangle \langle 0|_{E}+(1-\eta _{AB})|\phi _+\rangle \langle \phi _+|_{BE}, \end{aligned}$$

which satisfies $\textrm{Tr}_E\sigma _{a,BE}=\Lambda ^{(A\rightarrow B)}(|\psi _a\rangle \langle \psi _a|)$. The procedure for producing the composite state in Eq. (5) is illustrated in Fig. 2. In this figure, Eve lets Alice’s state be transmitted to Bob with a probability $\eta _{AB}$, or discard Alice’s state and share $|\phi _+\rangle$ with Bob with a probability $1-\eta _{AB}$. Let us suppose that $|\phi _+\rangle \langle \phi _+|$ is replaced to $\frac{1}{2}|11\rangle \langle 11|+\frac{1}{2}|22\rangle \langle 22|$ in Eq. (5), which means that Bob and Eve eventually shares a fully separable state. In this case, each Kraus operator of Bob transforms $\frac{1}{2}|11\rangle \langle 11|+\frac{1}{2}|22\rangle \langle 22|$ to another rank-2 state. It leads us to that Eve fails to design a quantum measurement used in type-I and -II eavesdropping schemes. Thus, Eve needs entanglement between herself and Bob in order to obtain meaningful information about his outcome.

These two types can provide same security. That is because the joint measurement probability between Bob and Eve in the type-I structure is equal to that in the type-II structure. Particularly, the type-II structure can be easily reproduced in an experimental setup.

### Sequential state discrimination including eavesdropper

For the security analysis, we propose the new sequential state discrimination for describing the two eavesdropper’s schemes. We first explain the structure of sequential state discrimination, and propose the optimal success probability of eavesdropping. We further investigate the amount of the secret key rate in frame of the sequential state discrimination scenario.

#### Structure of sequential state discrimination

Let us first explain how each of the eavesdropping scheme introduced in the previous section is described as a sequential state discrimination problem. It is noted that the unambiguous discrimination can be applied to the B92 protocol^3,58^. For this reason, we consider that Bob has a quantum measurement which can unambiguously discriminates Alice’s states $|\psi _0\rangle$ and $|\psi _1\rangle$.

We first consider the type-I structure. We note in advance that our argument in here can also be applied to the type-II structure. Suppose that positive-operator valued measure (POVM) $\{M_0^{(B)},M_1^{(B)},M_?^{(B)}\}$ denotes the measurements of Bob. Then, the Kraus operator $K_b^{(B)}$ corresponding to the POVM element $M_b^{(B)}$ ($b\in \{0,1,?\}$) is given by^34,39,40^:

6 $$\begin{aligned}{}&K_0^{(B)}=\sqrt{\alpha _0}|\phi _0^{(B)}\rangle \langle \alpha _0|, \ \ K_1^{(B)}=\sqrt{\alpha _1}|\phi _1^{(B)}\rangle \langle \alpha _1|,\nonumber \\&K_?^{(B)}=\sqrt{1-\alpha _0}|\phi _0^{(B)}\rangle \langle \alpha _0|+\sqrt{1-\alpha _1}|\phi _1^{(B)}\rangle \langle \alpha _1|. \end{aligned}$$

Here, $\alpha _0$ and $\alpha _1$ are non-negative parameters^40^, and $|\alpha _0\rangle$ and $|\alpha _1\rangle$ are corresponding vectors:

7 $$\begin{aligned} |\alpha _0\rangle= & {} \frac{1}{\sqrt{2(1+s)}}|1\rangle +\frac{1}{\sqrt{2(1-s)}}|2\rangle ,\nonumber \\ |\alpha _1\rangle= & {} \frac{1}{\sqrt{2(1+s)}}|1\rangle -\frac{1}{\sqrt{2(1-s)}}|2\rangle . \end{aligned}$$

For $a\not =b$, the inner product between $|\alpha _b\rangle$ and $|\psi _a\rangle$ is equal to zero. It guides us to the fact that the measurement described in terms of the Kraus operators in Eq. (6) can perform the unambiguous discrimination. When Bob obtains a conclusive result $b\in \{0,1\}$, the Kraus operator $K_b^{(B)}$ probabilistically changes the bipartite state of Eq. (3) into the following form:

8 $$\begin{aligned} K_0^{(B)}\otimes \mathbb {I}_E|\Gamma _0\rangle _{BE}= & \,|\phi _0^{(B)}\rangle _B\otimes |\gamma _{00}\rangle ,\nonumber \\ K_1^{(B)}\otimes \mathbb {I}_E|\Gamma _0\rangle _{BE}= & \,|\phi _1^{(B)}\rangle _B\otimes |\gamma _{01}\rangle ,\nonumber \\ K_0^{(B)}\otimes \mathbb {I}_E|\Gamma _1\rangle _{BE}= & \,|\phi _0^{(B)}\rangle _B\otimes |\gamma _{10}\rangle ,\nonumber \\ K_1^{(B)}\otimes \mathbb {I}_E|\Gamma _1\rangle _{BE}= & \,|\phi _1^{(B)}\rangle _B\otimes |\gamma _{11}\rangle ,\nonumber \\ \end{aligned}$$

where $|\gamma _{ab}\rangle$ are written as

9 $$\begin{aligned} |\gamma _{00}\rangle= & \,\mathscr {N}\left\{ \sqrt{\eta _{AB}\alpha _0}|0\rangle _E+\sqrt{\frac{(1-\eta _{AB})\alpha _0}{2(1-s^2)}}|\widetilde{\psi }_0\rangle _E\right\} ,\nonumber \\ |\gamma _{01}\rangle= & \,|\widetilde{\psi }_1\rangle _E,\nonumber \\ |\gamma _{10}\rangle= & \,|\widetilde{\psi }_0\rangle _E,\nonumber \\ |\gamma _{11}\rangle= & \,\mathscr {N}\left\{ \sqrt{\eta _{AB}\alpha _1}|0\rangle _E+\sqrt{\frac{(1-\eta _{AB})\alpha _1}{2(1-s^2)}}|\widetilde{\psi }_1\rangle _E\right\} . \end{aligned}$$

Here, $\mathscr {N}$ is the normalization constant and

10 $$\begin{aligned} |\widetilde{\psi }_b\rangle =\sqrt{1-s^2}|\alpha _b\rangle \end{aligned}$$

is a pure state spanned by $\{|1\rangle ,|2\rangle \}$. According to Eq. (10), $|\widetilde{\psi }_b\rangle$ is orthogonal to $|0\rangle$. Moreover, the label of $|\widetilde{\psi }_b\rangle$ in Eq. (8) is equal to the measurement result of Bob. Therefore, Eve can eavesdrop the measurement result of Bob by discriminating $|\widetilde{\psi }_0\rangle$ and $|\widetilde{\psi }_1\rangle$ with her measurement described as the POVM $\{M_0^{(E)},M_1^{(E)},M_?^{(E)}\}$ on the subspace spanned by $\{|1\rangle ,|2\rangle \}$,

11 $$\begin{aligned} M_0^{(E)}= & \,u_0|u_0\rangle \langle u_0|, \nonumber \\ M_1^{(E)}= & \,u_1|u_1\rangle \langle u_1|, \nonumber \\ M_?^{(E)}= & \, \mathbb {I}_E-M_0^{(E)}-M_1^{(E)}, \end{aligned}$$

where $M_e^{(E)}$ is the POVM element corresponding to the measurement result *e*. In Eq. (11), $\mathbb {I}_E$ is the identity operator on Eve’s system, $u_e$ is the non-negative real number, and $|u_e\rangle$ is the vector in the subspace $\{|1\rangle ,|2\rangle \}$ satisfying $\langle \widetilde{\psi }_b|u_e\rangle =\delta _{be}$. We note that $|u_e\rangle$ can be constructed in the same way as Eq. (7)^40^.

In the aspect of the quantum state discrimination task, the finite (but nonzero) success probability implies that a receiver can obtain an information about sender’s state^3^. Thus, one of the probable figures of merit is “the success probability of eavesdropping” in case of type-I structure, which is described as (the detailed evaluation is presented in Methods)

12 $$\begin{aligned} P_{s,\mathrm {type-I}}^{(E)}=\sum _{a,b\in \{0,1\}}q_a\langle \Gamma _a|K_b^{(B)\dagger }K_b^{(B)}\otimes \mathbb {I}_E|\Gamma _a\rangle \langle \gamma _{ab}|M_b^{(E)}|\gamma _{ab}\rangle . \end{aligned}$$

Assume that Bob performs optimal unambiguous discrimination on Alice’s state. Then, $P_{s,opt}^{(E)}$, which is the optimum success probability of eavesdropping, can have a simple expression such as $P_{s,opt1}^{(E)}$ or $P_{s,opt2}^{(E)}$,

13 $$\begin{aligned} P_{s,opt}^{(E)}= & \,\frac{1-\eta _{AB}}{2(1-s^2)}(\alpha _0+\alpha _1-2\sqrt{\alpha _0\alpha _1}s), \ \ \textrm{if} \ \ f_0(s)>0 \ \ \textrm{and} \ \ f_1(s)>0,\nonumber \\ P_{s,opt}^{(E)}= & \, \frac{1-\eta _{AB}}{2}\max \{\alpha _0,\alpha _1\}, \ \ \textrm{if} \ \ f_0(s)\le 0 \ \ \textrm{or} \ \ f_1(s)\le 0, \end{aligned}$$

with $$s:=|\langle \psi _1|\psi _2\rangle |$$ and

14 $$\begin{aligned}{} & {} f_0(s):=q_1s^3-\sqrt{q_0q_1}s^2-q_0s+\sqrt{q_0q_1},\nonumber \\{} & {} f_1(s):=q_0s^3-\sqrt{q_0q_1}s^2-q_1s+\sqrt{q_0q_1}. \end{aligned}$$

The detailed evaluation of the optimization is presented in Methods. If $s\in [0,\sqrt{q_1/q_2}]$, we get $\alpha _0=1-\sqrt{\frac{q_1}{q_0}}s$ and $\alpha _1=1-\sqrt{\frac{q_0}{q_1}}s$ from Bob’s optimal POVM condition^18^.

**Figure 3:**
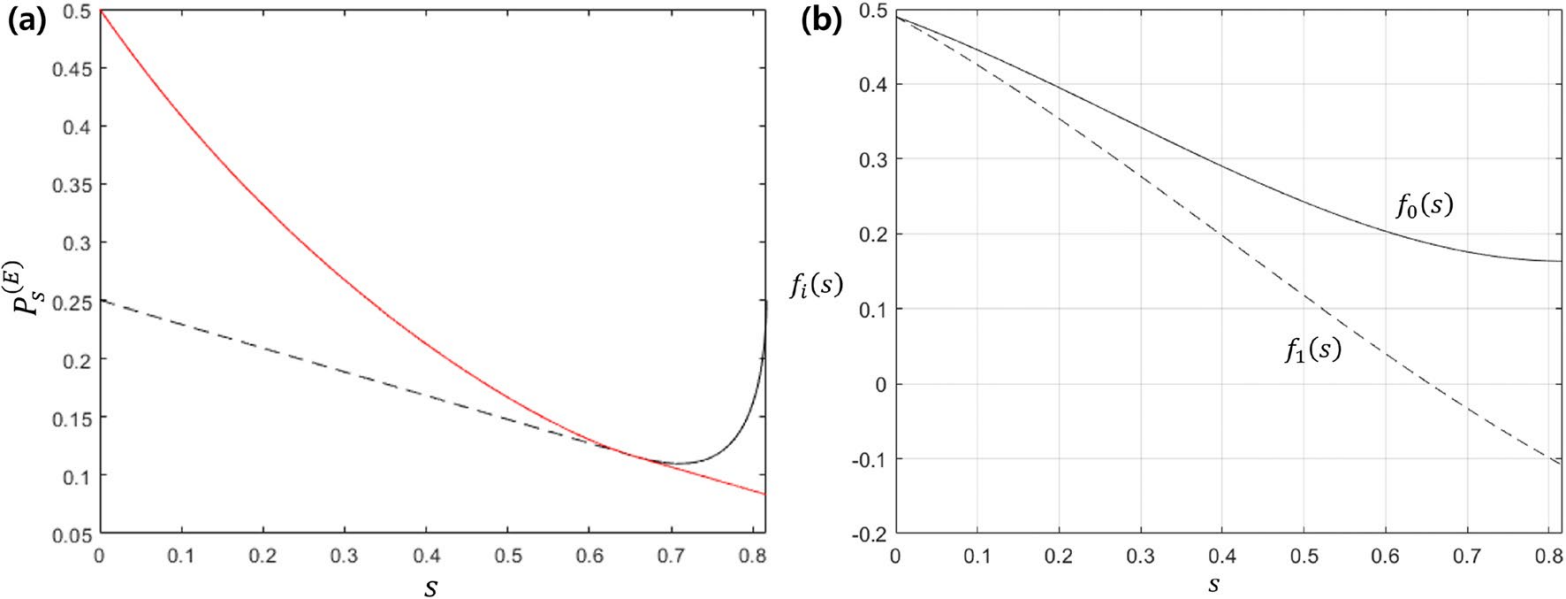
(**a**) Success probability of eavesdropping with respect to overlap $s=|\langle \psi _0|\psi _1\rangle |$ between two Alice’s states. Solid black line and dashed black line are success probabilities of eavesdropping $P_{s,opt1}^{(E)}$ and $P_{s,opt2}^{(E)}$ in Eq. (13), respectively, and solid red line is the optimal success probability of eavesdropping. In (**b**), $f_0(s)$ and $f_1(s)$ in Eq. (14) are depicted, where these functions are used for deciding which value between $P_{s,opt1}^{(E)}$ and $P_{s,opt2}^{(E)}$ is indeed optimal, on the basis of the condition written in Eq. (13).

Figure 3a illustrates the optimum success probability of eavesdropping($P_{s,opt}^{(E)}$) in Eq. (13). Here, we have used $q_0=0.4(q_1=0.6)$ and $\eta _{AB}=0.5$. In Fig. 3a, the solid black line(dashed black line) indicates $P_{s,opt1}^{(E)}$ ($P_{s,opt2}^{(E)}$). According to Fig. 3a, in the region of $s<0.6538$, $P_{s,opt1}^{(E)}$ (solid black line) is optimum. That is because, as illustrated in Fig. 3b, both $f_0(s)$ and $f_1(s)$ in Eq. (14) are non-negative in this region. Meanwhile, $P_{s,opt2}^{(E)}$ (dashed black line) is optimum in the region of $s>0.6538$, since one of $f_1(s)$ is negative. Thus, the optimum success probability of eavesdropping is indicated by the solid red line.

We further evaluate the success probability of eavesdropping in type-II structure as

15 $$\begin{aligned} P_{s,\mathrm {type-II}}^{(E)}=\sum _{a,b\in \{0,1\}}q_a\textrm{tr}\left[ K_b^{(B)}\otimes \mathbb {I}_E\sigma _{a,BE}K_b^{(B)\dagger }\otimes \mathbb {I}_E\right] \textrm{tr}\left[ \tau _{ab,E}M_b^{(E)}\right] , \end{aligned}$$

where $\tau _{ab,E}$ are defined as

16 $$\begin{aligned}{} & {} \tau _{00,E}=\frac{\eta _{AB}}{\eta _{AB}+\frac{1-\eta _{AB}}{2(1-s^2)}}|0\rangle \langle 0|_E+\frac{\frac{1-\eta _{AB}}{2(1-s^2)}}{\eta _{AB}+\frac{1-\eta _{AB}}{2(1-s^2)}}|\widetilde{\psi }_0\rangle \langle \widetilde{\psi }_0|_E,\nonumber \\{} & {} \tau _{01,E}=|\widetilde{\psi }_1\rangle \langle \widetilde{\psi }_1|_E,\nonumber \\{} & {} \tau _{10,E}=|\widetilde{\psi }_0\rangle \langle \widetilde{\psi }_0|_E,\nonumber \\{} & {} \tau _{11,E}=\frac{\eta _{AB}}{\eta _{AB}+\frac{1-\eta _{AB}}{2(1-s^2)}}|0\rangle \langle 0|_E+\frac{\frac{1-\eta _{AB}}{2(1-s^2)}}{\eta _{AB}+\frac{1-\eta _{AB}}{2(1-s^2)}}|\widetilde{\psi }_1\rangle \langle \widetilde{\psi }_1|_E. \end{aligned}$$

From the straightforward calculation, the success probability of eavesdropping in Eq. (15) is equal to Eq. (12). The proof is presented in Methods. Thus, the optimal success probability of eavesdropping in type-II structure is also analytically derived as Eq. (13).

**Figure 4:**
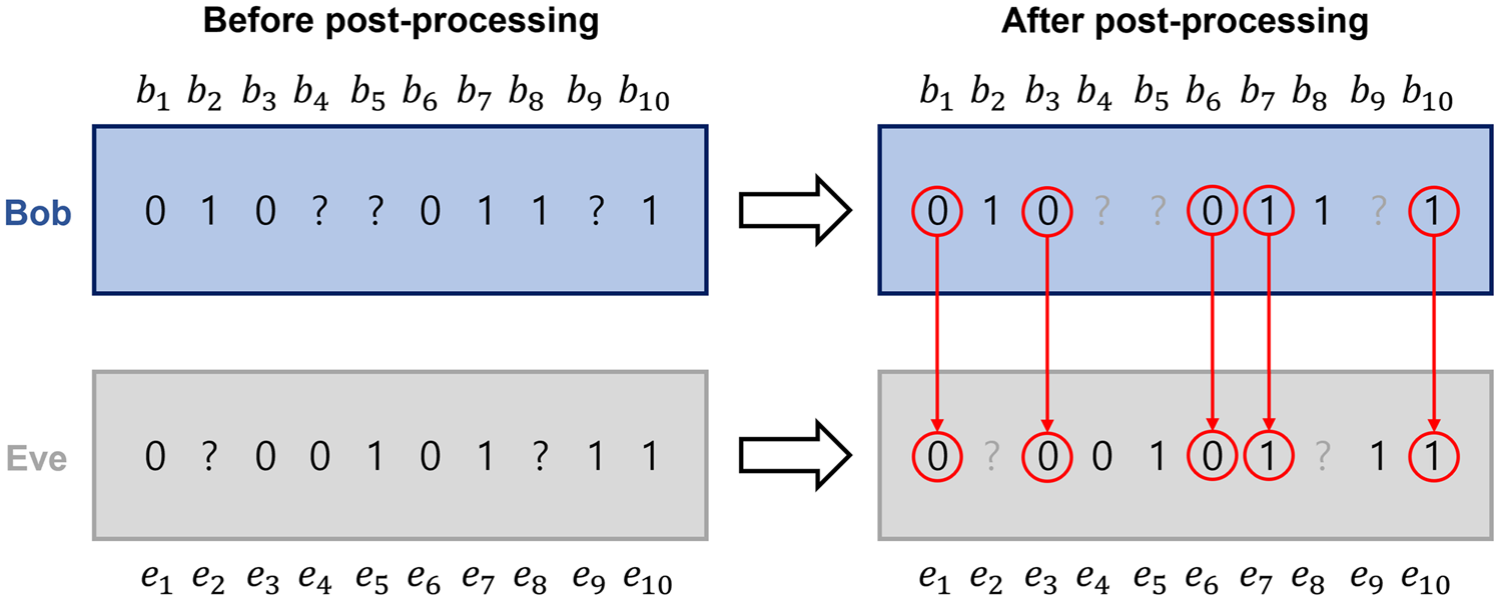
Post-processing performed by Bob and Eve. Let us suppose that Bob has 10 measurement results $b_1,\cdots ,b_{10}$, and Eve has measurement results $e_1,\cdots ,e_{10}$. Bob can discard the inconclusive results $b_4,b_5,b_{10}$, and Eve can also discard $e_2$ and $e_8$. We note that there may no classical communication between Bob and Eve. In other words, Eve does not have ability to discard her messages by presuming Bob’s inconclusive results. This supports the reason that the joint probability needs to be considered as Eq. (19).

#### Secret key rate

In sequential state discrimination scenario among Alice, Bob, and Eve, Alice and Bob can obtain secret key as follows. Let us suppose that Eve performs the eavesdropping scheme discussed in the previous section with optimal success probability of the eavesdropping. Then, due to Eve’s measurement which extracts information of Bob’s measurement outcome, an event that Alice’s prepared bit and Bob’s outcome are not equal happens with nonzero probability. By this discrepancy between Alice and Bob, they notice the presence of Eve. This means that Alice and Bob can share the secret key even though Eve performs most efficient eavesdropping scheme. Note that Bob performs optimal unambiguous discrimination on Alice’s states, he is not supposed to get error outcomes if Eve does not exist between Alice and Bob.

According to Csiszar and Korner^54^, when the amount of information between a receiver and a sender is larger than that between a receiver and eavesdropper, a secret key can exist as an amount equal to the difference of information. The secret key rate is defined as

17 $$\begin{aligned} K_{AB:E}= & {} \max \{0,I(B:A)-I(B:E)\}\nonumber \\= & {} \max \{0,H(A)-H(B,A)-H(E)+H(B,E)\}. \end{aligned}$$

Here, $I(X:Y)=H(X)+H(Y)-H(X,Y)$ is Shannon mutual information. *H*(*X*) denotes Shannon entropy and *H*(*X*, *Y*) is Shannon joint entropy. If $K_{AB:E}>0$, sender Alice and receiver Bob can share the secret key^54^.

As illustrated in Fig. 4, Bob and Eve can perform the following post-processing. In case that Bob performs optimal unambiguous discrimination, he can discard the measurement result when he obtains an inconclusive result. This post-processing can enhance the amount of information shared between Alice and Bob^59^. In this way, the joint probability between Alice and Bob is

18 $$\begin{aligned} \widetilde{P}_{AB}(a,b)=\frac{P_{AB}(a,b)}{\sum _{a,b\in \{0,1\}}P_{AB}(a,b)}, \end{aligned}$$

which constitutes the Shannon mutual information in Eq. (17). Here. $a,b\in \{0,1,?\}$ are the measurement results for Alice and Bob, respectively. Similarly, when Eve obtains an inconclusive result, she discards the measurement result. Thus, it seems that Eve can successfully obtain information about Bob. However, Bob and Eve are separated in space and the information leakage discussed above is not permitted. In other words, Eve cannot discard her measurement result based on whether Bob obtained an inconclusive result or not. Therefore, the joint probability between Bob and Eve should be changed as follows:

19 $$\begin{aligned} \widetilde{P}_{BE}(b,e)=\frac{P_{BE}(b,e)}{\sum _{b\in \{0,1,?\}}\sum _{e\in \{0,1\}}P_{BE}(b,e)}, \end{aligned}$$

where $b,e\in \{0,1,?\}$ are the measurement results for Bob and Eve, respectively.

**Figure 5:**
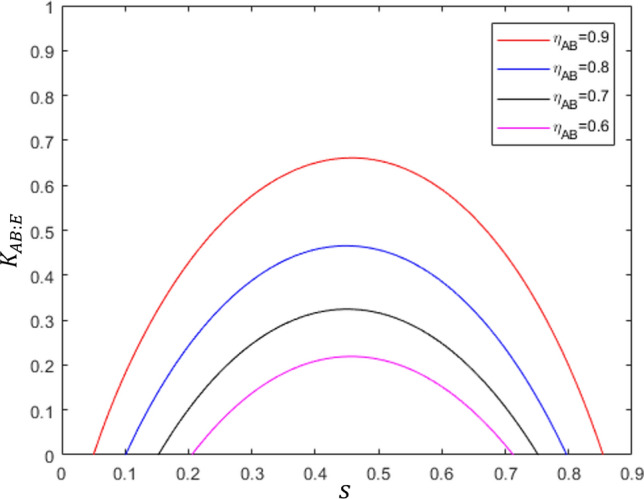
Secret key rate $K_{AB:E}$: red, blue, black, and purple lines correspond to $\eta _{AB}=0.9$, $\eta _{AB}=0.8$, $\eta _{AB}=0.7$, and $\eta _{AB}=0.6$, respectively. Here, *s* is the overlap between two Alice’s states.

Figure 5 shows the secret key rate $K_{AB:E}$ written in Eq. (17), considering the marginal probability between Bob and Eve which is updated from Eq. (19). Here, $P_{AB}(a,b)$ and $P_{BE}(b,e)$ in Eqs. (18) and (19) are evaluated by considering Bob’s POVM optimizing optimal unambiguous discrimination and Eve’s POVM maximizing success probability of eavesdropping (For the details, see “Secret key rate” in Methods). We note that the both two types of eavesdropper’s scheme provides same secret key rate (for detail, see Methods). Here, the channel efficiency is considered as $\eta _{AB}=0.9$(solid red line), $\eta _{AB}=0.8$(solid blue line), $\eta _{AB}=0.7$(solid black line), and $\eta _{AB}=0.6$(solid purple line). As shown in Fig. 5, as the overlap *s* increases, $K_{AB:E}$ also increases. However, from a specific overlap $K_{AB:E}$ decreases. For example, for $\eta _{AB}=0.9$, in the region of $s<0.4585$, $K_{AB:E}$ increases but in the region of $s>0.4585$, $K_{AB:E}$ decreases.

The secret key rate $K_{AB:E}$ exhibits interesting behavior. When the overlap *s* is large, it is difficult for Bob and Eve to efficiently implement the quantum state discrimination. In this case, the mutual information between Alice and Bob, and Bob and Eve becomes small. However, when *s* is small, Bob and Eve can easily and efficiently implement the quantum state discrimination. In this case, the mutual information between Alice and Bob, and Bob and Eve becomes large.

**Figure 6:**
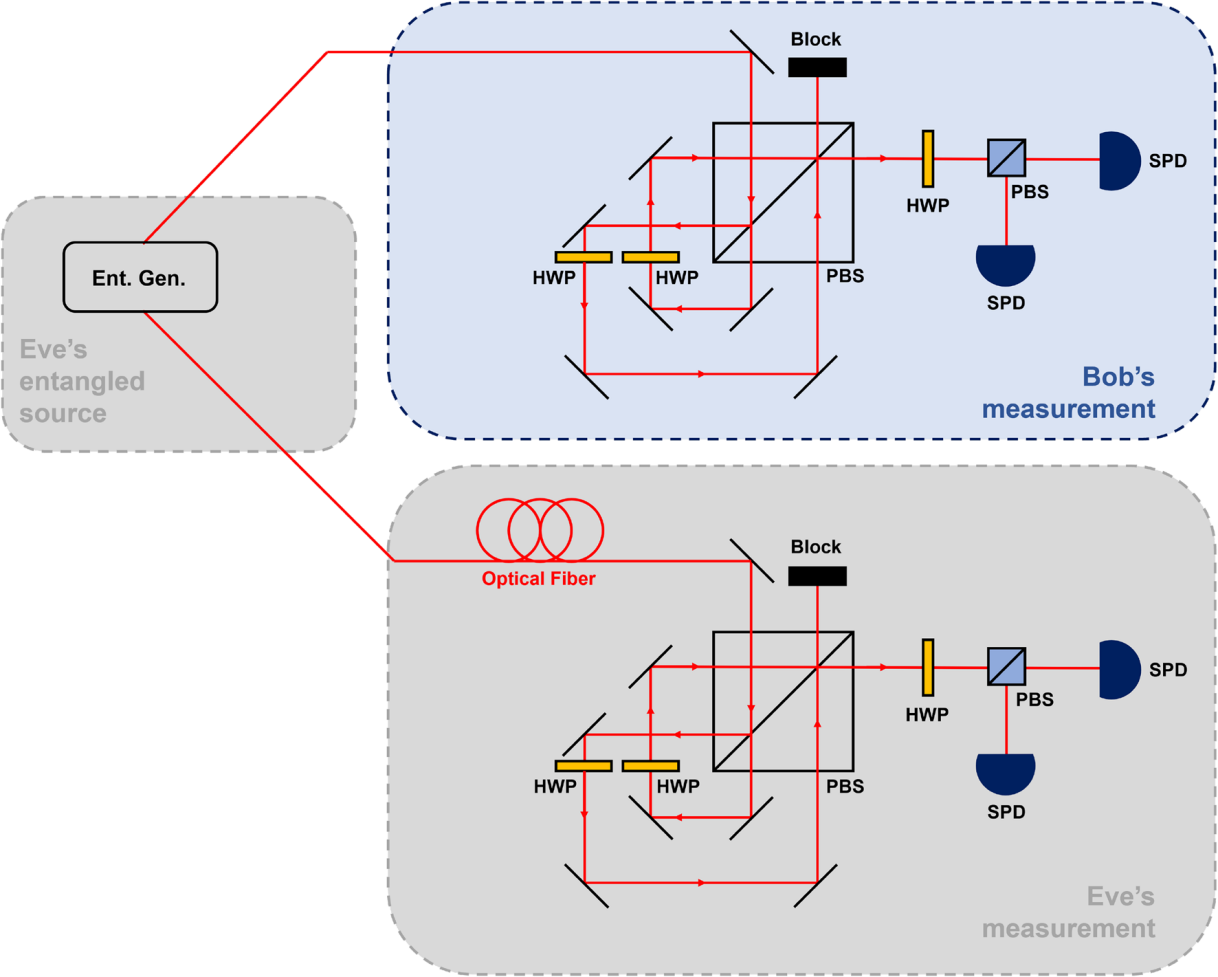
Experimental setting for implementing type-II structure of eavesdropping. Here, with probability $1-\eta _{AB}$, Eve discards Alice’s state and prepares maximally entangled state $|\phi _+\rangle =\frac{1}{\sqrt{2}}(|hh\rangle +|vv\rangle )$ between Eve and Bob. HWP: half-wave plate, PBS: polarized beam splitter, SPD: single-photon detector, and Ent. Gen.: entanglement generator^60^.

### Method for experimental implementation

Let us propose an experimental method for a unified model of sequential state discrimination including an eavesdropper with quantum optics. Even though the type-I structure was used previously, we will use type-II structure, because it can be easily implemented in an experimental setup. In the type-II structure, Alice prepares a quantum state

20 $$\begin{aligned} |\psi _a\rangle =\sqrt{\frac{1+s}{2}}|h\rangle +(-1)^a\sqrt{\frac{1-s}{2}}|v\rangle , \end{aligned}$$

where $|h\rangle$ and $|v\rangle$ represent horizontal and vertical directions, respectively. Eve, who controls channel efficiency $\eta _{AB}$, can eavesdrop as follows: (i) With a probability of $\eta _{AB}$, Eve does not eavesdrop on the quantum state of Alice. (ii) With a probability of $1-\eta _{AB}$, Eve eliminates the quantum state of Alice and shares a maximally entangled state with Bob. (iii) After Bob’s measurement, Eve performs measurement on her subsystem.

In Fig. 6 of the next page, we illustrate the experimental setup(for details about the description, see Supplementary information). Here, the experimental setup of Bob and Eve is based on a Sagnac-like interferometer^55^. The setup consists of a half-wave plate(HWP), polarized beam splitter(PBS), and single-photon detector(SPD). In step (ii), Eve generates a maximally entangled two-photon polarization state $|\phi _+\rangle =\frac{1}{\sqrt{2}}(|hh\rangle +|vv\rangle )$, using a type-II spontaneous parametric down conversion(SPDC)^60^. Type-II SPDC includes beta-barium borate(BBO) crystals, two birefringent crystals, HWP, and quarter-wave plate(QWP). HWP and QWP transform the entangled pure state, generated by the BBO and birefringent crystals, into one of the four Bell-states. Note that the maximally entangled two-photon polarization state is also efficiently generated by the Sagnac interferometer in which a periodically-poled KTP crystal is equipped.

According to the type-II structure, if Eve generates $|\phi _+\rangle$ with a probability of $1-\eta _{AB}$, Eve can eavesdrop on the result of Bob, based on the selection of the path of a single photon and the measurement result of two SPDs. Ideally, Bob performs an unambiguous discrimination based on a Sagnac-like interferometer, and Eve can eavesdrop with the optimum success probability of eavesdropping by constructing a Sagnac-like interferometer. It should be emphasized that despite the attack by Eve, Alice and Bob can obtain the secret key rate.

In reality, one should consider imperfections occurring in the photon state and in SPD. We consider the dark count rate($\nu >0$) and detection efficiency($0<\eta <1$) for the SPD. The photon state in the setup consists of two types: a single-photon polarization state that Alice sends to Bob, and the single photon state of maximally entangled state generated by Eve. Different types of photon states suffer from different types of noises. For example, the single-photon polarization state may disappear under a noisy channel, which is called “amplitude damping”^61,62^. We assume that amplitude damping can occur between Alice and Bob and between Bob and Eve. In addition, white or colored noise can occur when Eve generates a maximally entangled quantum state^56^. Particularly, colored noise which occurs because of imperfections in experimental entangling operations is more frequent than white noise^56^. By including all the imperfections discussed above, Bob and Eve eventually shares the following quantum state:

21 $$\begin{aligned} \zeta _{a}=\eta _{AB}\Lambda _{D_0}^{(ad)}(|\psi _a\rangle \langle \psi _a|)_B\otimes |0\rangle \langle 0|_E+(1-\eta _{AB})\left( \Lambda _{D_e}^{(ad)}\otimes \Lambda _{D_e}^{(ad)}\right) (\varrho _{ent}), \end{aligned}$$

for given Alice’s bit $a\in \{0,1\}$. Here, $\eta _{AB}$ is the channel efficiency between Alice and Bob, $\Lambda _{D_0}^{(ad)}$ is an amplitude damping channel between Alice and Bob with damping ratio $D_0$, and $\Lambda _{D}^{(ad)}$ is the amplitude damping channel between Eve and Bob with the damping ratio $D_e$. $\varrho _{ent}$ is a noisy entangled state generated by Eve. If Eve’s entangled state is exposed to white noise, then the noisy entangled state is written as^56^

22 $$\begin{aligned} \rho _{ent}^{(wh)}=\eta _{\textrm{ent}} |\phi _+\rangle \langle \phi _+|+(1-\eta _{\textrm{ent}})\frac{1}{4}(|h\rangle \langle h|+|v\rangle \langle v|)\otimes (|h\rangle \langle h|+|v\rangle \langle v|), \end{aligned}$$

and if it is exposed to color noise, then^56^

23 $$  \rho _{{ent}}^{{(cl)}}  = \eta _{{ent}}|\phi _{ + } \rangle \langle \phi _{ + } | + (1 - \eta _{{{\text{ent}}}} )\frac{1}{2}(|hh\rangle \langle hh| + |vv\rangle \langle vv|), $$

with the efficiency of entanglement $\eta _{ent}$, where $|\phi _+\rangle =(|hh\rangle +|vv\rangle )/\sqrt{2}$ is a maximally entangled state composed of horizontal and vertical states $|h\rangle$ and $|v\rangle$.

**Figure 7:**
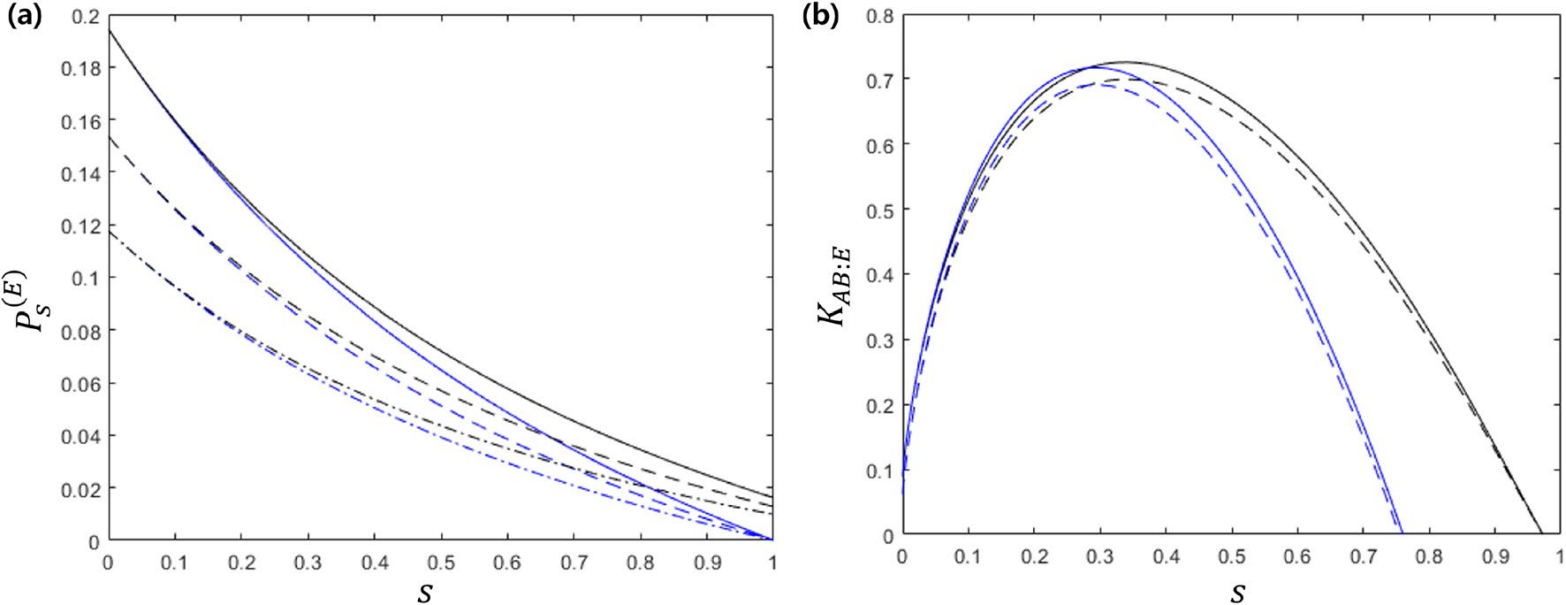
(**a**) Success probability of eavesdropping under imperfect quantum channel, entangled state, and single-photon detector. (**b**) Secret key rate between Alice and Bob. Here, $\eta _{AB}$, $\eta _{ent}$, and $\eta$ in Eq. (21) are $\eta _{AB}=0.5$, $\eta _{ent}=0.5$, and $\eta =0.8$, respectively. Blue(black) line corresponds to color(white) noise. Solid, dashed, and dash-dot lines correspond to $D=0.1$, $D=0.2$, and $D=0.3$, respectively. Here, we assume that $D_0=D_e=D$ for considering the relation between the secret key rate and a single decoherence parameter. Secret key rate under imperfect quantum channel, entangled state, and single-photon detector. Here, $\eta _{AB}=0.5$, $\eta _{ent}=0.5$, and $\eta =0.8$ are considered. Blue(black) line corresponds to color(white) noise. Solid(dashed) line corresponds to $D_0=0.1$($D_0=0.2$). In every case, $D_e=0.4$ is considered.

The success probability of eavesdropping under imperfections described as Eqs. (21)–(23) is displayed in Fig. 7a (for detail, see Supplementary information). In Fig. 7a, the value of $\eta _{AB}=0.5$, $\eta _{ent}=0.5$, and $\eta =0.8$ are considered, where the detection efficiency $\eta =0.8$ is the value of a commercialized superconducting nanowire single-photon detector(SNSPD) whose dark count rate is nearly zero^63^. In Fig. 7a, the solid line, dashed line, and dash-dot line correspond to the cases of decoherence parameter, $D=0.1$, $D=0.2$, and $D=0.3$, respectively(a large *D* implies that the decoherence rate is high). Here, we assume that $D_0=D_e=D$ for considering the relation between the secret key rate and a single decoherence parameter. The black and blue lines show the cases of white and colored noise, respectively.

In Fig. 7b, the secret key rate between Alice and Bob with the imperfections in Eqs. (21)–(23) is displayed, considering various imperfections (for detail, see Supplementary information). Here, $\eta _{AB}=0.5$, $\eta _{ent}=0.5$, and $\eta =0.8$ are considered. The blue(black) line corresponds to colored(white) noise. The solid(dashed) line corresponds to $D_0=0.1$($D_0=0.2$). In every case, $D_e$ is taken as 0.4. It should be noted that the secret key rate does not change when $D_0=D_e$ owing to the post-processing expressed in Eq. (19). As shown in Fig. 7b, the graph of the secret key rate has one global maximum. This implies that (i) if *s* tends to be smaller, then the secret key rate decreases because the tendency of *s* makes Eve as well as Bob to easily discriminate the quantum states, and (ii) if *s* tends to be larger, then the secret key rate decreases because the tendency of *s* makes discrimination performed by Bob and Eve difficult.

## Conclusion

In this paper, we have proposed a unified model of sequential state discrimination including an eavesdropper. We have shown that even though Eve uses an entanglement to eavesdrop on Bob’s measurement result, Alice and Bob can have a non-zero secret key rate. Furthermore, we have proposed an experimental model for eavesdropping. Because our experimental method consists of linear optical technologies, the implementation of our method is practical. Ideally, our experiment can achieve optimum success probability of eavesdropping. Beyond the ideal case, we have investigated possible imperfections including quantum channels between Alice and Bob, entanglement between Bob and Eve, and the inefficiency of Bob’s SPD. It is interesting that the non-zero secret key rate is possible even under such the imperfections.

In this paper, we have focused on security analysis of the B92 protocol in view of the sequential state discrimination scheme. That is because the security analysis can be performed with the simple mathematical structure of the unambiguous discrimination^22,40^. We emphasize that our methodology based on the sequential state discrimination can be applied to the various kinds of quantum communication^64^ as well as quantum key distribution^45,65^ designed in prepare-and-measure way. Moreover, our scheme can be applied to quantum communication or key distribution task utilizing the continuous variable quantum systems^57,66^. We further emphasize that our research propose a novel theoretical way to unify the secure quantum communication tasks in terms of the quantum state discrimination.

It also should be noted that our sequential state discrimination model can be extended to the case of unambiguously discriminating *N* pure states^1,22^. This extension is important since large *N* guarantees large amount of transmitted bits per a signal pulse. Moreover, our experimental idea can also be applied to the continuous variable version. That is because sequential measurement that unambiguously discriminates two coherent states can be designed with linear optics^41^.

## Methods

### Success probability of eavesdropping

In this section, we derive and optimize the success probability of eavesdropping by considering both types of eavesdropping strategies.

#### Describing type-I structure of eavesdropper

In this structure, the following entangled state between Bob and Eve is considered:

24 $$\begin{aligned} |\Gamma _a\rangle _{BE}=\sqrt{\eta _{AB}}|\psi _a\rangle _B\otimes |0\rangle _E+\sqrt{\frac{1-\eta _{AB}}{2}}(|11\rangle +|22\rangle )_{BE}. \end{aligned}$$

Then, each $K_b^{(B)}\otimes \mathbb {I}_E|\Gamma _a\rangle$ are obtained by

25 $$\begin{aligned} K_0^{(B)}\otimes \mathbb {I}_E|\Gamma _0\rangle= & {} \sqrt{\eta _{AB}\alpha _0}|\phi _0^{(B)}\rangle _B\otimes |0\rangle _E\nonumber +\sqrt{\frac{(1-\eta _{AB})\alpha _0}{2}} \left\{ (|\phi _0^{(B)}\rangle \langle \alpha _0|\otimes \mathbb {I}_E)|11\rangle +(|\phi _0^{(B)}\rangle \langle \alpha _0|\otimes \mathbb {I}_E)|22\rangle \right\} _{BE},\nonumber \\ K_1^{(B)}\otimes \mathbb {I}_E|\Gamma _0\rangle= & {} \sqrt{\frac{(1-\eta _{AB})\alpha _1}{2}}\left\{ (|\phi _1^{(B)}\rangle \langle \alpha _1|\otimes \mathbb {I}_E)|11\rangle +(|\phi _1^{(B)}\rangle \langle \alpha _1|\otimes \mathbb {I}_E)|22\rangle \right\} _{BE},\nonumber \\ K_0^{(B)}\otimes \mathbb {I}_E|\Gamma _1\rangle= & {} \sqrt{\frac{(1-\eta _{AB})\alpha _0}{2}} \left\{ (|\phi _0^{(B)}\rangle \langle \alpha _0|\otimes \mathbb {I}_E)|11\rangle +(|\phi _0^{(B)}\rangle \langle \alpha _0|\otimes \mathbb {I}_E)|22\rangle \right\} _{BE},\nonumber \\ K_1^{(B)}\otimes \mathbb {I}_E|\Gamma _1\rangle= & {} \sqrt{\eta _{AB}\alpha _1}|\phi _1^{(B)}\rangle _B\otimes |0\rangle _E +\sqrt{\frac{(1-\eta _{AB})\alpha _1}{2}}\left\{ (|\phi _1^{(B)}\rangle \langle \alpha _1|\otimes \mathbb {I}_E)|11\rangle +(|\phi _1^{(B)}\rangle \langle \alpha _1|\otimes \mathbb {I}_E)|22\rangle \right\} _{BE}. \end{aligned}$$

Without loss of generality, we write pure states $|\psi _a\rangle$ as

26 $$\begin{aligned} |\psi _0\rangle _E= & {} \sqrt{\frac{1+s}{2}}|1\rangle _E+\sqrt{\frac{1-s}{2}}|2\rangle _E,\nonumber \\ |\psi _1\rangle _E= & {} \sqrt{\frac{1+s}{2}}|1\rangle _E-\sqrt{\frac{1-s}{2}}|2\rangle _E, \end{aligned}$$

and vectors $|\alpha _b\rangle$ in the Kraus operators as

27 $$\begin{aligned} |\alpha _0\rangle _E= & {} \frac{1}{\sqrt{2(1+s)}}|1\rangle _E+\frac{1}{\sqrt{2(1-s)}}|2\rangle _E,\nonumber \\ |\alpha _1\rangle _E= & {} \frac{1}{\sqrt{2(1+s)}}|1\rangle _E-\frac{1}{\sqrt{2(1-s)}}|2\rangle _E, \end{aligned}$$

such that $\langle \psi _a|\alpha _b\rangle =\delta _{ab}$ for every $a,b\in \{0,1\}$. Substituting Eqs. (26) and (27) into Eq. (25), we obtain

28 $$\begin{aligned} K_0^{(B)}\otimes \mathbb {I}_E|\Gamma _0\rangle= & {} \sqrt{\eta _{AB}\alpha _0}|\phi _0^{(B)}\rangle _B\otimes |0\rangle _E+\sqrt{\frac{(1-\eta _{AB})\alpha _0}{2}}|\phi _0^{(B)}\rangle _B\otimes |\alpha _0\rangle _E,\nonumber \\ K_1^{(B)}\otimes \mathbb {I}_E|\Gamma _0\rangle= & {} \sqrt{\frac{(1-\eta _{AB})\alpha _1}{2}}|\phi _1^{(B)}\rangle _B\otimes |\alpha _1\rangle _E,\nonumber \\ K_0^{(B)}\otimes \mathbb {I}_E|\Gamma _1\rangle= & {} \sqrt{\frac{(1-\eta _{AB})\alpha _0}{2}}|\phi _0^{(B)}\rangle _B\otimes |\alpha _0\rangle _E,\nonumber \\ K_1^{(B)}\otimes \mathbb {I}_E|\Gamma _1\rangle= & {} \sqrt{\eta _{AB}\alpha _1}|\phi _1^{(B)}\rangle _B\otimes |0\rangle _E+\sqrt{\frac{(1-\eta _{AB})\alpha _1}{2}}|\phi _1^{(B)}\rangle _B\otimes |\alpha _1\rangle _E. \end{aligned}$$

We define (normalized) pure states by

29 $$\begin{aligned} |\widetilde{\psi }_a\rangle _E=\sqrt{1-s^2}|\alpha _a\rangle _E. \end{aligned}$$

Substituting Eq. (29) into Eq. (28), we obtain the representation of Eq. (6) in the letter with $|\gamma _{ab}\rangle$ defined by

30 $$\begin{aligned} |\gamma _{00}\rangle _E= & {} \frac{1}{\sqrt{\eta _{AB}+\frac{(1-\eta _{AB})}{2(1-s^2)}}}\left\{ \sqrt{\eta _{AB}}|0\rangle +\sqrt{\frac{(1-\eta _{AB})}{2(1-s^2)}}|\widetilde{\psi }_0\rangle \right\} _E,\nonumber \\ |\gamma _{01}\rangle _E= & {} |\widetilde{\psi }_1\rangle _E,\nonumber \\ |\gamma _{10}\rangle _E= & {} |\widetilde{\psi }_0\rangle _E,\nonumber \\ |\gamma _{11}\rangle _E= & {} \frac{1}{\sqrt{\eta _{AB}+\frac{(1-\eta _{AB})}{2(1-s^2)}}}\left\{ \sqrt{\eta _{AB}}|0\rangle +\sqrt{\frac{(1-\eta _{AB})}{2(1-s^2)}}|\widetilde{\psi }_1\rangle \right\} _E. \end{aligned}$$

Consider Eve’s POVM as $\{M_0^{(E)},M_1^{(E)},M_?^{(E)}\}$ where each POVM element is given by

31 $$\begin{aligned} M_0^{(E)}=u_0|u_0\rangle \langle u_0|, \ \ M_1^{(E)}=u_1|u_1\rangle \langle u_1|, \ \ M_?^{(E)}=\mathbb {I}_E-M_0^{(E)}-M_1^{(E)}, \end{aligned}$$

where $u_0$ and $u_1$ are non-negative real numbers, and $|u_0\rangle$ and $|u_1\rangle$ are vectors orthogonal to $|0\rangle _E$ and satisfying $\langle u_b|\widetilde{\psi }_a\rangle =\delta _{ab}$ for every $a,b\in \{0,1\}$.

From Eqs. (28)–(31), the success probability of eavesdropping is obtained by

32 $$\begin{aligned} P_s^{(E)}=\frac{1-\eta _{AB}}{2(1-s^2)}(\alpha _0u_0+\alpha _1u_1). \end{aligned}$$

#### Optimization

According to Eq. (29), inner product $\langle \widetilde{\psi }_0|\widetilde{\psi }_1\rangle$ is obtained by

33 $$\begin{aligned} \langle \widetilde{\psi }_0|\widetilde{\psi }_1\rangle =-s. \end{aligned}$$

Since $|u_0\rangle \bot |0\rangle _E$ and $|u_1\rangle \bot |0\rangle _E$, supports of $M_0^{(E)}$ and $M_1^{(E)}$ are also orthogonal to $|0\rangle _E$. This implies that Eve’s POVM is designed to discriminate $|\widetilde{\psi }_0\rangle$ and $|\widetilde{\psi }_1\rangle$. Therefore, the constraint of Eve’s POVM is given by^39^

34 $$\begin{aligned} (1-u_0)(1-u_1)\ge s^2. \end{aligned}$$

Therefore, we obtain the following optimization problem:

35 $$\begin{aligned} \textrm{maximize }{} & {} \ \ P_s^{(E)}=\frac{1-\eta _{AB}}{2(1-s^2)}(\alpha _0u_0+\alpha _1u_1),\nonumber \\ \mathrm {subject \ to }{} & {} \ \ (1-u_0)(1-u_1)\ge s^2. \end{aligned}$$

For fixed parameters $\alpha _0$ and $\alpha _1$, an optimal point $(u_0,u_1)$ satisfies

36 $$\begin{aligned} (1-u_0)(1-u_1)=s^2. \end{aligned}$$

Also, for the optimal point, there exists a non-zero real number $\lambda$ satisfying

37 $$\begin{aligned}  \overrightarrow {\nabla }  P_s^{(E)}=\lambda  \overrightarrow {\nabla }  \left\{ (1-u_0)(1-u_1)-s^2\right\} , \end{aligned}$$

where $\overrightarrow {\nabla }$ is a gradient such that $\overrightarrow {\nabla }  f=\left( \frac{\partial f}{\partial u_0},\frac{\partial f}{\partial u_1}\right)$. We note that Eq. (37) is equivalent to^39^

38 $$\begin{aligned} \frac{\partial P_s^{(E)}/\partial u_0}{\partial P_s^{(E)}/\partial u_1}=\frac{\partial \left\{ (1-u_0)(1-u_1)-s^2\right\} /\partial u_0}{\partial \left\{ (1-u_0)(1-u_1)-s^2\right\} /\partial u_1}. \end{aligned}$$

Combining Eqs. (36) and (38), we obtain the optimal point by

39 $$\begin{aligned} u_0=1-\sqrt{\frac{\alpha _1}{\alpha _0}}s, \ \ u_1=1-\sqrt{\frac{\alpha _0}{\alpha _1}}s. \end{aligned}$$

Since the optimal point $(u_0,u_1)$ is on the surface of Eq. (34), both $u_0$ and $u_1$ in Eq. (39) should be non-negative. For this reason, the overlap *s* also should be

40 $$\begin{aligned} s<\sqrt{\frac{\alpha _0}{\alpha _1}} \ \wedge \ s<\sqrt{\frac{\alpha _1}{\alpha _0}}. \end{aligned}$$

Considering *s* in the region of Eq. (40), the optimal success probability of eavesdropping is analytically given by

41 $$\begin{aligned} P_{s,opt1}^{(E)}=\frac{1-\eta _{AB}}{2(1-s^2)}(\alpha _0+\alpha _1-2\sqrt{\alpha _0\alpha _1}s). \end{aligned}$$

Suppose that Bob performs optimal unambiguous discrimination between two pure states $|\psi _0\rangle$ and $|\psi _1\rangle$. Then, $\alpha _0$ and $\alpha _1$ are given by^18^

42 $$\begin{aligned} \alpha _0=1-\sqrt{\frac{q_1}{q_0}}s, \ \ \alpha _1=1-\sqrt{\frac{q_0}{q_1}}s, \end{aligned}$$

if

43 $$\begin{aligned} s<\sqrt{\frac{q_1}{q_0}} \ \wedge \ s<\sqrt{\frac{q_0}{q_1}}. \end{aligned}$$

Substituting Eq. (42) with $\alpha _0$ and $\alpha _1$ in Eq. (40), we obtain

44 $$\begin{aligned}{} & {} f_0(s):=q_1s^3-\sqrt{q_0q_1}s^2-q_0s+\sqrt{q_0q_1}>0,\nonumber \\{} & {} f_1(s):=q_0s^3-\sqrt{q_0q_1}s^2-q_1s+\sqrt{q_0q_1}>0. \end{aligned}$$

We note that if one of inequalities in Eq. (44) does not hold, then the optimal point $(u_0,u_1)$ is given by

45 $$\begin{aligned} (u_0,u_1)\in \{(1,0),(0,1)\}. \end{aligned}$$

Substituting this optimal point into Eq. (32), we obtain the optimal success probability of eavesdropping:

46 $$\begin{aligned} P_{s,opt2}^{(E)}=\frac{1-\eta _{AB}}{2}\max \{\alpha _0,\alpha _1\}. \end{aligned}$$

#### Describing type-II structure of eavesdropper’s scheme

In this structure, the following bipartite state between Bob and Eve is considered:

47 $$\begin{aligned} \sigma _{a,BE}=\eta _{AB}|\psi _a\rangle \langle \psi _a|_B\otimes |0\rangle \langle 0|_E+(1-\eta _{AB})|\phi _+\rangle \langle \phi _+|_{BE}. \end{aligned}$$

Then, each $K_b^{(B)}\otimes \mathbb {I}_E\sigma _{a,AB}K_b^{(B)\dagger }\otimes \mathbb {I}_E$ is obtained by

48 $$\begin{aligned} K_0\otimes \mathbb {I}_E\sigma _{0,BE}K_0^{\dagger }\otimes \mathbb {I}_E= & \, \eta _{AB}\alpha _0|\phi _0^{(B)}\rangle \langle \phi _0^{(B)}|\otimes |0\rangle \langle 0|_E+\frac{(1-\eta _{AB})\alpha _0}{2(1-s^2)}|\phi _0^{(B)}\rangle \langle \phi _0^{(B)}|\otimes |\widetilde{\psi }_0\rangle \langle \widetilde{\psi }_0|_E,\nonumber \\ K_1\otimes \mathbb {I}_E\sigma _{0,BE}K_1^{\dagger }\otimes \mathbb {I}_E= & \, \frac{(1-\eta _{AB})\alpha _1}{2(1-s^2)}|\phi _1^{(B)}\rangle \langle \phi _1^{(B)}|\otimes |\widetilde{\psi }_1\rangle \langle \widetilde{\psi }_1|_E,\nonumber \\ K_0\otimes \mathbb {I}_E\sigma _{1,BE}K_0^{\dagger }\otimes \mathbb {I}_E= & \, \frac{(1-\eta _{AB})\alpha _0}{2(1-s^2)}|\phi _0^{(B)}\rangle \langle \phi _0^{(B)}|\otimes |\widetilde{\psi }_0\rangle \langle \widetilde{\psi }_0|_E,\nonumber \\ K_1\otimes \mathbb {I}_E\sigma _{1,BE}K_1^{\dagger }\otimes \mathbb {I}_E= & \, \eta _{AB}\alpha _1|\phi _1^{(B)}\rangle \langle \phi _1^{(B)}|\otimes |0\rangle \langle 0|_E+\frac{(1-\eta _{AB})\alpha _1}{2(1-s^2)}|\phi _1^{(B)}\rangle \langle \phi _1^{(B)}|\otimes |\widetilde{\psi }_1\rangle \langle \widetilde{\psi }_1|_E. \end{aligned}$$

We derive the success probability of eavesdropping as

49 $$\begin{aligned} P_s^{(E)}=\sum _{a,b\in \{0,1\}}q_a\textrm{tr}\left[ K_b^{(B)}\otimes \mathbb {I}_E\sigma _{a,BE}K_b^{(B)\dagger }\otimes \mathbb {I}_E\right] \textrm{tr}\left[ \tau _{ab,E}M_b^{(E)}\right] , \end{aligned}$$

where $\tau _{ab,E}$ are defined as

50 $$\begin{aligned}{} & {} \tau _{00,E}=\frac{\eta _{AB}}{\eta _{AB}+\frac{1-\eta _{AB}}{2(1-s^2)}}|0\rangle \langle 0|_E+\frac{\frac{1-\eta _{AB}}{2(1-s^2)}}{\eta _{AB}+\frac{1-\eta _{AB}}{2(1-s^2)}}|\widetilde{\psi }_0\rangle \langle \widetilde{\psi }_0|_E,\nonumber \\{} & {} \tau _{01,E}=|\widetilde{\psi }_1\rangle \langle \widetilde{\psi }_1|_E,\nonumber \\{} & {} \tau _{10,E}=|\widetilde{\psi }_0\rangle \langle \widetilde{\psi }_0|_E,\nonumber \\{} & {} \tau _{11,E}=\frac{\eta _{AB}}{\eta _{AB}+\frac{1-\eta _{AB}}{2(1-s^2)}}|0\rangle \langle 0|_E+\frac{\frac{1-\eta _{AB}}{2(1-s^2)}}{\eta _{AB}+\frac{1-\eta _{AB}}{2(1-s^2)}}|\widetilde{\psi }_1\rangle \langle \widetilde{\psi }_1|_E. \end{aligned}$$

From Eqs. (48) and (50), the success probability of eavesdropping in Eq. (49) is obtained by Eq. (32).

### Secret key rate

In this section, we derive the secret key rate when Eve’s POVM optimizes the success probability of eavesdropping.

#### Secret key rate of type-I eavesdropping structure

To derive the secret key rate, we need to evaluate entropies *H*(*A*), *H*(*B*, *A*), *H*(*E*) and *H*(*B*, *E*). For equal prior probabilities (*i.e*., $q_0=q_1$), *H*(*A*) is given by

51 $$\begin{aligned} H(A)=-q_0\log _2q_0-q_1\log _2q_1=1. \end{aligned}$$

Also, *H*(*B*, *A*) is given by

52 $$\begin{aligned} H(B,A)=-\sum _{a,b\in \{0,1\}}\widetilde{P}_{AB}(a,b)\log _2\widetilde{P}_{AB}(a,b), \end{aligned}$$

where $\widetilde{P}_{AB}(a,b)$ is a post-processed joint probability between Alice and Bob after Bob discards his inconclusive result:

53 $$\begin{aligned} \widetilde{P}_{AB}(a,b)=\frac{P_{AB}(a,b)}{\sum _{a,b\in \{0,1\}}P_{AB}(a,b)}, \end{aligned}$$

and $P_{AB}(a,b)$ is a pre-processed joint probability

54 $$\begin{aligned} P_{AB}(a,b)=q_a\textrm{tr}\left\{ \Lambda ^{(A\rightarrow B)}(|\psi _a\rangle \langle \psi _a|)K_b^{(B)\dagger }K_b^{(B)}\right\} =\frac{1}{2}\left\{ \eta _{AB}\alpha _b\delta _{ab}+\frac{(1-\eta _{AB})\alpha _b}{2(1-s^2)}\right\} . \end{aligned}$$

Here, we consider the post-processing that Bob discard his inconclusive result, since this post-processing can enhance unambiguous quantum communication protocol^59^.

Since $q_0=q_1$ implies $u_0=u_1=1-s$ according to Eqs. (39) and (42), the joint probability of Eq. (54) is rewritten by

55 $$\begin{aligned} P_{AB}(a,b)= & {} \frac{1}{2}\left\{ \eta _{AB}(1-s)+\frac{1-\eta _{AB}}{2(1+s)}\right\} , \ \ \textrm{if} \ \ (a,b)\in \{(0,0),(1,1)\}, \end{aligned}$$

56 $$\begin{aligned} P_{AB}(a,b)= & {} \frac{1-\eta _{AB}}{4(1+s)}, \ \ \textrm{if} \ \ (a,b)\in \{(0,1),(1,0)\}. \end{aligned}$$

To evaluate *H*(*B*, *E*) and *H*(*E*), we first consider a joint probability $P_{ABE}(a,b,e)$ among Alice, Bob and Eve:

57 $$\begin{aligned} P_{ABE}(a,b,e)=P_{AB}(a,b)P_{E|AB}(e|a,b)=q_aP_{B|A}(b|a)P_{E|AB}(e|a,b), \end{aligned}$$

where $P_{B|A}(b|a)$ and $P_{E|AB}(e|a,b)$ are conditional probabilities.

1. In case of $b\not =?$, every $|\gamma _{ab}\rangle$ in Eq. (30) is rewritten by
  58 $$\begin{aligned} |\gamma _{ab}\rangle _E=\frac{1}{\sqrt{P_{B|A}(b|a)}}\left\{ \sqrt{\eta _{AB}\alpha _b}\delta _{ab}|0\rangle +\sqrt{\frac{(1-\eta _{AB})\alpha _b}{2(1-s^2)}}|\widetilde{\psi }_b\rangle \right\} _E, \end{aligned}$$
  where
  59 $$\begin{aligned} P_{B|A}(b|a)=\eta _{AB}\alpha _b\delta _{ab}+\frac{(1-\eta _{AB})\alpha _b}{2(1-s^2)}. \end{aligned}$$
  Therefore, $P_{E|AB}(e|a,b)$ is given by
  60 $$\begin{aligned} P_{E|AB}(e|a,b)=\langle \gamma _{ab}|M_e^{(E)}|\gamma _{ab}\rangle =\frac{1}{P_{B|A}(b|a)}\frac{(1-\eta _{AB})\alpha _b}{2(1-s^2)}u_e\delta _{be}. \end{aligned}$$
  Substituting Eq. (60) into $P_{E|AB}(e|a,b)$ of Eq. (57), we obtain
  61 $$\begin{aligned} P_{ABE}(a,b,e)=q_a\frac{(1-\eta _{AB})\alpha _b}{2(1-s^2)}u_e\delta _{be}, \end{aligned}$$
  and
  62 $$\begin{aligned} P_{BE}(b,e)=\sum _{a=0}^{1}P_{ABE}(a,b,e)=\frac{(1-\eta _{AB})\alpha _b}{2(1-s^2)}u_e\delta _{be}, \end{aligned}$$
2. in case of $b=?$, we provide following equality:
  63 $$\begin{aligned} K_?^{(B)}\otimes \mathbb {I}_E|\Gamma _a\rangle \nonumber =&\sqrt{\eta _{AB}(1-\alpha _0)}\delta _{a0}|\phi _0^{(B)}\rangle _B\otimes |0\rangle _E+\sqrt{\frac{(1-\eta _{AB})(1-\alpha _0)}{2(1-s^2)}}|\phi _0^{(B)}\rangle _B\otimes |\widetilde{\psi }_0\rangle _E,\nonumber \\ &  +\sqrt{\eta _{AB}(1-\alpha _1)}\delta _{a1}|\phi _1^{(B)}\rangle _B\otimes |0\rangle _E+\sqrt{\frac{(1-\eta _{AB})(1-\alpha _1)}{2(1-s^2)}}|\phi _1^{(B)}\rangle _B\otimes |\widetilde{\psi }_1\rangle _E. \end{aligned}$$
  In the same way as Eq. (30), we obtain
  64 $$\begin{aligned} |\gamma _{a?}\rangle _E=\frac{1}{P_{B|A}(?|a)}\sum _{x\in \{0,1\}}\left\{ \sqrt{\eta _{AB}(1-\alpha _x)}\delta _{ax}|0\rangle +\sqrt{\frac{(1-\eta _{AB})(1-\alpha _x)}{2(1-s^2)}}|\widetilde{\psi }_x\rangle \right\} _E. \end{aligned}$$
  (Since $P_{B|A}(?|a)$ is too complicated, we do not describe it in detail.) Therefore, $P_{E|AB}(e|a,?)$ is given by
  65 $$\begin{aligned} P_{E|AB}(e|a,?)=\frac{1}{P_{B|A}(?|a)}\left\{ \frac{(1-\eta _{AB})(1-\alpha _0)}{2(1-s^2)}u_e\delta _{e0}+\frac{(1-\eta _{AB})(1-\alpha _1)}{2(1-s^2)}u_e\delta _{e1}\right\} . \end{aligned}$$
  Substituting Eq. () into $P_{E|AB}(e|a,b)$ of Eq. (57), we obtain
  66 $$\begin{aligned} P_{ABE}(a,b,e)=q_a\left\{ \frac{(1-\eta _{AB})(1-\alpha _0)}{2(1-s^2)}u_e\delta _{0e}+\frac{(1-\eta _{AB})(1-\alpha _1)}{2(1-s^2)}u_e\delta _{1e}\right\} , \end{aligned}$$
  and
  67 $$\begin{aligned} P_{BE}(?,e)=\sum _{a\in \{0,1\}}P_{ABE}(a,?,e)=\frac{(1-\eta _{AB})(1-\alpha _0)}{2(1-s^2)}u_e\delta _{0e}+\frac{(1-\eta _{AB})(1-\alpha _1)}{2(1-s^2)}u_e\delta _{1e}, \end{aligned}$$

Since $q_0=q_1$ implies $u_0=u_1=1-s$ according to Eqs. (39) and (42), the joint probability $P_{BE}(b,e)$ of Eqs. (62) and (67) are rewritten by

68 $$\begin{aligned} P_{BE}(b,e)= & {} \frac{(1-\eta _{AB})(1-s)}{2(1+s)}, \ \ \textrm{if} \ \ (b,e)\in \{(0,0),(1,1)\},\nonumber \\ P_{BE}(b,e)= & {} 0, \ \ \textrm{if}\hspace{5.0pt}(b,e)\in \{(0,1),(1,0)\},\nonumber \\ P_{BE}(b,e)= & {} \frac{(1-\eta _{AB})s}{2(1+s)}, \ \ \textrm{if} \ \ (b,e)\in \{(0,?),(1,?)\}. \end{aligned}$$

If Eve discard her inconclusive result, the post-processed joint probability is given by

69 $$\begin{aligned} \widetilde{P}_{BE}(b,e)=\frac{P_{BE}(b,e)}{\sum _{b\in \{0,1,?\}}\sum _{e\in \{0,1\}}P_{BE}(b,e)}, \end{aligned}$$

and a marginal probability $\widetilde{P}_E(e)$ is given by

70 $$\begin{aligned} \widetilde{P}_E(e)=\sum _{b\in \{0,1,?\}}\widetilde{P}_{BE}(b,e). \end{aligned}$$

Finally, *H*(*E*) and *H*(*B*, *E*) are evaluated as

71 $$\begin{aligned} H(E)= & {} -\sum _{e\in \{0,1\}}\widetilde{P}_{E}(e)\log _2\widetilde{P}_{E}(e),\nonumber \\ H(B,E)= & {} -\sum _{b\in \{0,1,?\}}\sum _{e\in \{0,1\}}\widetilde{P}_{BE}(b,e)\log _2\widetilde{P}_{BE}(b,e). \end{aligned}$$

#### Secret key rate of type-II eavesdropping structure

We first note that the prior probabilities and the quantum channel $\Lambda ^{(A\rightarrow B)}$ are invariant under the choice of structure. Therefore, *H*(*A*) and *H*(*B*, *A*) are evaluated as Eqs. (51) and (52) in the type-I structure.

1. In case of $b\not =?$, every $\tau _{ab,E}$ in Eq. (50) is rewritten by
  72 $$\begin{aligned} \tau _{ab,E}=\frac{1}{P_{B|A}(b|a)}\left\{ \eta _{AB}\alpha _b\delta _{ab}|0\rangle \langle 0|+\frac{(1-\eta _{AB})\alpha _b}{2(1-s^2)}|\widetilde{\psi }_b\rangle \langle \widetilde{\psi }_b|\right\} , \end{aligned}$$
  where $P_{B|A}(b|a)$ is given by Eq. (59). Moreover, $P_{E|AB}(e|a,b)$ is given by
  73 $$\begin{aligned} P_{E|AB}(e|a,b)=\textrm{tr}\left\{ \tau _{ab,E}M_e^{(E)}\right\} =\frac{1}{P_{B|A}(b|a)}\frac{(1-\eta _{AB})\alpha _b}{2(1-s^2)}u_e\delta _{be}, \end{aligned}$$
  which is equal to Eq. (60). Therefore, according to Eq. (57), $P_{ABE}(a,b,e)$ *is equal to the case of type-I structure.*
2. In case of $b=?$, we consider
  74 $$\begin{aligned} K_?^{(B)}\otimes \mathbb {I}_E\sigma _{a,BE}K_?^{(B)}\otimes \mathbb {I}_E= & {} \eta _{AB}\Gamma (\sqrt{1-\alpha _0}\delta _{a0}|\phi _0^{(B)}\rangle +\sqrt{1-\alpha _1}\delta _{a1}|\phi _1^{(B)}\rangle )\otimes |0\rangle \langle 0|_E\nonumber \\+ & {} (1-\eta _{AB})\Gamma \left( \sqrt{\frac{1-\alpha _0}{2(1-s^2)}}|\phi _0^{(B)}\rangle \otimes |\widetilde{\psi }_0\rangle +\sqrt{\frac{1-\alpha _1}{2(1-s^2)}}|\phi _1^{(B)}\rangle \otimes |\widetilde{\psi }_1\rangle \right) , \end{aligned}$$
  where we define $\Gamma (|v\rangle ):=|v\rangle \langle v|$ for convenience. From the above representation, we define a bipartite mixed state shared by Bob and Eve:
  75 $$\begin{aligned} \tau _{a?,BE}= & \,\frac{1}{P_{B|A}(b|a)}\Big [\eta _{AB}\Gamma (\sqrt{1-\alpha _0}\delta _{a0}|\phi _0^{(B)}\rangle +\sqrt{1-\alpha _1}\delta _{a1}|\phi _1^{(B)}\rangle )\otimes |0\rangle \langle 0|_E\nonumber \\ &+ (1-\eta _{AB})\Gamma \left( \sqrt{\frac{1-\alpha _0}{2(1-s^2)}}|\phi _0^{(B)}\rangle \otimes |\widetilde{\psi }_0\rangle +\sqrt{\frac{1-\alpha _1}{2(1-s^2)}}|\phi _1^{(B)}\rangle \otimes |\widetilde{\psi }_1\rangle \right) \Big ].\nonumber \\ \end{aligned}$$
  Then, $P_{E|AB}(e|a,?)$ is given by
  76 $$\begin{aligned} P_{E|AB}(e|a,?)= & \,\textrm{tr}\left\{ \tau _{a?,BE}\left( \mathbb {I}_B\otimes M_e^{(E)}\right) \right\} \nonumber \\= & {} \frac{1}{P_{B|A}(?|a)}\left\{ \frac{(1-\eta _{AB})(1-\alpha _0)}{2(1-s^2)}u_e\delta _{e0}+\frac{(1-\eta _{AB})(1-\alpha _1)}{2(1-s^2)}u_e\delta _{e1}\right\} .\nonumber \\ \end{aligned}$$
  This is equal to Eq. (65), which implies that $P_{A,B,E}(a,b,?)$ *is also equal to the case of type-I structure.*

From the above calculation, we confirm that *H*(*B*, *E*) and *H*(*E*) in this structure is equal to these in the type-I structure, respectively. This leads us to the result that both structures provide same secret key rate.

It is noted that the formalism of the joint probabilities discussed above can also provide the success probability of eavesdropping. This will be further explained in the next section.

#### Revisiting success probability of eavesdropping in terms of joint probabilities

In the scenario of the new sequential discrimination, Bob’s measurement result *b* depends on the input *a* prepared by Alice, and Eve’s measurement result *e* depends on *a* and *b*. From these facts, the joint probability between three parties $P_{ABE}(a,b,e)$ is easily derived by

77 $$\begin{aligned} P_{ABE}(a,b,e)=q_aP_{B|A}(b|a)P_{E|AB}(e|a,b)=q_aP_{BE|A}(b,e|a), \end{aligned}$$

where $q_a$ is the prior probability that Alice prepares *a*, $P_{B|A}(b|a)$ is the conditional probability that Bob obtains *b* if Alice prepares *a*, $P_{E|AB(e|a,b}$ is the conditional probability that Eve obtains *e* if Alice prepares *a* and Bob obtains *b*, $P_{BE|A}(b,e|a$ is the conditional joint probability that Bob and Eve obtain *b* and *e* if Alice prepares *a*, and $P_{BE}(b,e)$ is the joint probability that Bob and Eve obtain *b* and *e*. Also, the success probability of eavesdropping is derived by

78 $$\begin{aligned} P_s^{(E)}=\sum _{a,b}q_aP_{B|A}(b|a)P_{E|AB}(e=b|a,b)=\sum _{a,b}q_aP_{BE|A}(b,e=b|a)=\sum _{b}P_{BE}(b,e=b). \end{aligned}$$

It is noted that the expression of the success probability of eavesdropping in Eq. (78) is used for deriving the success probability of eavesdropping when Alice, Bob, and Eve performs the scenario by using the imperfect linear optical technologies.

## Data Availability

Te datasets used and analysed during the current study available from the corresponding author on reasonable request.
